# Severe and Moderate Asthma Exacerbations in Asthmatic Children and Exposure to Ambient Air Pollutants

**DOI:** 10.3390/ijerph13080771

**Published:** 2016-08-01

**Authors:** Louis-Francois Tétreault, Marieve Doucet, Philippe Gamache, Michel Fournier, Allan Brand, Tom Kosatsky, Audrey Smargiassi

**Affiliations:** 1 Department of Environmental and Occupational Health, School of Public Health, University of Montreal, Montréal, QC H2L 1M3, Canada; louis-francois.tetreault@umontreal.ca; 2 Direction Régionale de Santé Publique du CIUSSS Centre-Sud-de-l’Île-de-Montréalde Montréal, Montréal, QC H2L 1M3, Canada; mfournie@santepub-mtl.qc.ca; 3 Institut Natonal de la Santé Publique du Québec, Québec, QC H2L 1M3, Canada; marieve.doucet.1@ulaval.ca (M.D.); philippe.gamache@inspq.qc.ca (P.G.); allan.brand83@gmail.com (A.B.); 4 Department of Medicine, Laval University, Québec, QC H2L 1M3, Canada; 5 British Columbia Centre for Disease Control, Vancouver, BC V5Z 4R4, Canada; tom.kosatsky@bccdc.ca; 6 Université de Montréal Public Health Research Institute, Montreal, QC H2L 1M3, Canada

**Keywords:** asthma, air pollution, childhood, exacerbation

## Abstract

Background: It is well established that short-term exposure to ambient air pollutants can exacerbate asthma, the role of early life or long-term exposure is less clear. We assessed the association between severe asthma exacerbations with both birth and annual exposure to outdoor air pollutants with a population-based cohort of asthmatic children in the province of Quebec (Canada). Method: Exacerbations of asthma occurring between 1 April 1996 and 31 March 2011 were defined as one hospitalization or emergency room visit with a diagnosis of asthma for children (<13 years old) already diagnosed with asthma. Annual daily average concentrations of ozone (O_3_) and nitrogen dioxide (NO_2_) were estimated at the child’s residential postal code. Satellite based levels of fine particulate (PM_2.5_) estimated for a grid of 10 km by 10 km were also assigned to postal codes of residence for the whole province. Hazard ratios (HRs) were estimated from Cox models with a gap time approach for both birth and time-dependant exposure. Results: Of the 162,752 asthmatic children followed (1,020,280 person-years), 35,229 had at least one asthma exacerbation. The HRs stratified by age groups and adjusted for the year of birth, the ordinal number of exacerbations, sex, as well as material and social deprivation, showed an interquartile range increase in the time-dependant exposure to NO_2_ (4.95 ppb), O_3_ (3.85 ppb), and PM_2.5_ (1.82 μg/m^3^) of 1.095 (95% CI 1.058–1.131), 1.052 (95% CI 1.037–1.066) and 1.025 (95% CI 1.017–1.031), respectively. While a positive association was found to PM_2.5_, no associations were found between exposure at birth to NO_2_ or O_3_. Conclusions: Our results support the conclusion, within the limitation of this study, that asthma exacerbations in asthmatic children are mainly associated with time dependent residential exposures less with exposure at birth.

## 1. Introduction

Asthma is one of the most common chronic diseases in childhood worldwide and its prevalence continues to increase in many developed countries [1,2]. Asthma exacerbation is a significant cause of hospitalizations and emergency room visits, especially for the minority of children with asthma who contribute to the majority of the health care service use [3,4,5]. Exacerbation is defined by the Global Initiative for Asthma group as an inflammation of the airways causing asthma symptoms (wheezing, breathlessness, chest tightness and coughing) by restricting or limiting the airflow to and from the lungs.

Even though the most frequent trigger of asthma exacerbations are respiratory tract infections; other factors, such as genetic predisposition and exposure to specific environmental risk factors, are also suspected to trigger asthma exacerbations [4,5,6]. Two of these factors are long-term exposure to environmental tobacco smoke [7,8,9] and short-term exposure to ambient air pollutants [10,11,12].

Studies that have assessed the effect of short term air pollution exposure and asthma exacerbation in asthmatic children, found consistent associations with every level of severity (mild, moderate and severe) of exacerbation [10,11,12]. Positive associations with severe exacerbations, defined as emergency department visits or hospitalization, were reported for daily levels of ozone (O_3_), nitrogen dioxide (NO_2_) and particulate matters (PM) [13,14,15,16].

Long-term childhood exposure to outdoor air pollution has also been linked to lung function decrease in asthmatic children [17]. Furthermore, studies conducted in Southern California have also shown that lung function growth was associated with long-term air pollution exposure in children [18,19]. However, only a restricted number of studies assessed the relation between long-term exposure to ambient air pollutants and asthma exacerbations in asthmatic children [20,21,22,23,24]. Of these studies, three [20,22,24] focused on severe exacerbations, and all three used traffic density as a proxy of traffic-related air pollution exposure. Only the study by Wilhelm et al., 2008, also presents associations between long-term time dependent exposure to O_3_ or PM [24].

In this study we assessed the relation between long-term time dependent (i.e., exposure based on yearly pollutant levels) or birth exposure to O_3_, NO_2_ and fine particulate matter (PM_2.5_) and severe exacerbation of asthma in asthmatic children identified from a population based birth cohort.

## 2. Methods

### 2.1. Population

We studied asthmatic children born between 1 April 1996 and 31 March 2011 in the province of Quebec (Canada) and identified from a population open birth cohort created with the Quebec Integrated Chronic Disease Surveillance System (QICDSS) [25]. Children younger than 13 years of age were included in this cohort after they were diagnosed as asthmatic based on administrative data. All asthmatic children were followed up to 31 March 2011. In the QICDSS, the health insurance registry, medical services (services dispensed through physician claims in emergency rooms and in physician’s office), hospital admissions and deaths are linked. The QICDSS contains information on health services for about 95% of the Quebec population [26]. The health insurance registry contains demographic information for all children born and living in Quebec and demographic information (birth date, sex, and residential six character postal code). When children move, parents or guardians submit their new address, but this is done on a voluntary basis. Additionally, a mandatory address update is required every four years.

In the QICDSS, new cases of asthma were identified (using codes of the International Classification of Diseases) either by one hospital discharge with a diagnosis of asthma (in any diagnostic field) or two physician claims for asthma (visits to the emergency room or physician’s office) occurring within a two years period.

### 2.2. Asthma Exacerbations

Asthma exacerbations were defined as either by a hospitalization admission or an emergency room visit for asthma in children already diagnosed with asthma. Following each exacerbation, we imposed a 30 days buffer period during which subjects were not considered at risk of another exacerbation. This buffer period was put in place in order to avoid counting a single asthmatic exacerbation a multiple number of times.

### 2.3. Estimation of Exposure

In order to assess the relation between birth and time dependent exposure to air pollutants and severe exacerbation of asthma in asthmatic children, we linked existing yearly average levels of NO_2_ (from 1996 to 2006, for the Montreal Island sub-cohort only) and O_3_ (from 1999 to 2011) to the centroid of the six-character postal code of the residence of the asthmatic children during the follow-up period. For PM_2.5_, only one value, i.e., the mean levels for 2001–2006 estimated for a 10 km × 10 km grid, was linked to each postal code (the same for each year from 1996 to 2011), which was assumed constant for the study period. Only a small decreasing trend in satellite derived annual PM_2.5_ levels has been noted during the study period [27]. Thus levels of air pollutants at a postal code centroid were applied to all residences in the postal code. In this study, exposure at birth was assigned according to the calendar year of the birth; thus, someone born 15 January or 30 October, 2010, were given the same exposure. When a child moved, its exposure was changed to reflect its new postal code from the day the address change took effect. In urban areas, six-character postal codes often corresponds to a single segment of road in which fewer than 50 individuals live; in rural areas, these postal codes can cover a large territory. Centroids for postal codes were taken from the Canada Postal Code Conversion Files [28].

Yearly average levels of air pollutants, based on the calendar year, were estimated at the residential six digit postal codes of the children with the following models. We used annual mean of daily NO_2_ levels (in ppb) estimated on a 5 m by 5 m grid for the island of Montreal (a sub-cohort of the Quebec cohort) with a Land Use Regression model developed by Crouse et al., 2009 [29]. Briefly, NO_2_ levels were measured in 133 locations on the island of Montreal with passive diffusion samplers during three seasons in 2005 and 2006. As reported by Crouse et al., 2009, NO_2_ levels for this period ranged from 2.6 to 31.5 ppb [29]. NO_2_ levels prior to 2005 were derived from a back-extrapolation of the model. The extrapolation method involved multiplying the modeled NO_2_ levels by the site specific ratio of past concentrations from fixed-site monitors of the Canadian National Air Pollution Surveillance (NAPS) network [30].

For each year, we estimated average summer O_3_ levels (in ppb) at the postal code of each child with a Bayesian Maximum Entropy model described in Adam-Poupart et al. (2014) [31]. Concisely, O_3_ levels were estimated using a geographical interpolation from a combination of measured levels at the NAPS stations in Quebec (1990–2009) and estimates from a land use mixed effect model. The land use mixed effect model was developed with the NAPS data and road network, meteorological data and latitude. We used summer O_3_ daily levels (in ppb) as proxy of yearly exposure to O_3_ levels.

We also used mean 2001–2006 PM_2.5_ levels derived from satellite imagery by van Donkelaar et al. (2010) with column aerosol optical depth (AOD) measurements using satellite instruments (Multiangle Imaging Spectroradiometer and Moderate Resolution Imaging Spectroradiometer) [32]. van Donkelaar et al. (2010) converted AOD measurements into surface PM_2.5_ levels using the global chemical transport model GEOS-Chem. Concentrations of PM_2.5_ (in µg/m^3^) were estimated for a grid of 10 km by 10 km and the value of each grid was assigned to all postal codes that were found in it [32]. For children who didn’t move during their follow-up, the birth and time dependent exposure were identical.

### 2.4. Socioeconomic Status

Because socio-economic status of subjects was not available on an individual-basis, we approximated the socio-economic status of the asthmatic children for each year of the study using an area-wide variable that represents “deprivation” [33]. This index, based on six indicators taken from the Canadian census, is divided into two components to assess material and social deprivation. Dissemination areas sometimes encompass a number of postal codes in the urban regions of our study. We assigned values of the 1996 census for the follow-up occurring before 1999, values of the 2001 census for the follow-up between 1999 and 2003 and the 2006 values after 2003.

### 2.5. Statistical Analysis

The linearity of the relations with continuous exposure variables was assessed using restricted cubic splines with three knots, with knots positioned at quantiles 0.10, 0.50 and 0.90 and the statistical significance of the non-linear terms was assessed with likelihood ratio tests at α of 0.05. The proportional hazard assumption was assessed through the examination of the weighted and scaled *Schoenfeld* residuals in order to assess evident trend with time [34].

For each pollutant we present crude associations from Cox models as well as two adjusted models. The first is adjusted for sex, year of birth and quintiles of the Pampalon deprivation indices. The second is adjusted for the same variables as the first model and for the ordinal number of recurring events. We used of the ordinal number of the recurring event (i.e., 1st, 2nd, etc.) to check if hazard rates were modified by the past history of exacerbations. Since age did not meet the proportional hazard assumption, we present associations stratified for age. The stratification was performed according to three age groups (<4, 4–8 and >8 years old).

Information on second hand smoke (SHS) exposure was unavailable for this cohort. Thus to control for the potential influence of SHS, we performed an indirect adjustment for the subgroup of the Montreal children, using a strategy proposed by Steenland and Greenland, 2004, and adapted by Villeneuve et al., 2011, for continuous exposures as a sensitivity analysis [35,36]. To perform this analysis, we used area-specific prevalence of at home childhood exposure to SHS obtained from a 2006 survey conducted in Montreal [37], as well as a rate ratio representing the association between childhood asthma and SHS [38]. This analysis is described in a previous study [25].

Furthermore, the following sensitivity analyses were performed: (1) excluding regions where health services may be under reported (i.e., where physicians receive a salary or mixed reimbursements for fee-for services and salary; where residents are likely to use the health care system of another province); (2) using subjects that did not move during their follow-up.

We also tested for effect modification between air pollutant exposure and deprivation with a product term between air pollutant levels and Pampalon indices or sex.

All analyses were performed with SAS 9.4 with the exception of the indirect adjustment for tobacco exposure that was performed with R (version 3.1.0 with packages mvtnorm and mcsm) [39]. The project was carried out in the context of the Quebec ministerial health surveillance plan. The Research Ethics Board of public health (ISBN: 978-2-550-58576-3, 14 of January 2010) and the “Commission d’accès à l’information” has approved the use of the QICDSS.

## 3. Results

The provincial cohort consisted in 162,752 asthmatic children. Asthmatic children had an average follow-up time of 6.25 years. As shown in Table 1, the majority of the follow-up (64.73%) occurred in children younger than five years old. In the cohort, 22.03% of the asthmatic children had at least one exacerbation (0.46 exacerbations on average) with a mean of 541 days before an exacerbation occurs (after the diagnosis or the previous exacerbation). Even though 99% of the asthmatic children had less than 10 exacerbations during their follow-up, the maximum number of exacerbations observed in an individual was 47.

There were 39,065 asthmatic children born on the Island of Montreal who constituted the subgroup studied for associations with NO_2_. As in the provincial cohort, most of the follow-up occurred in children younger than five years old (70.01%) but the average follow-up time was longer (6.53 years). In this subgroup, the proportion of asthmatic children with at least one exacerbation (27.14%) as well as the average number of exacerbation per children (0.66) was higher than in the provincial cohort (Table 1).

Distributions of NO_2_, PM_2.5_ and O_3_ at the birth residential postal code and at the time dependent residential postal code during the follow-up were similar (Table 2). The average exposure levels to NO_2_ (for the Montreal sub-cohort) were slightly higher at the birth than for the time dependent levels (respectively, 15.51 and 15.04 ppb) whereas the opposite was observed for O_3_ (respectively, 29.78 and 30.57 ppb). Finally the time dependent and birth exposures remained practically identical for PM_2.5_. We found moderate inverse correlations over the years between O_3_ and NO_2_, as well as between O_3_ and PM_2.5_ (range 0.25–0.61). Inversely, we found a moderate positive correlation between NO_2_ and PM_2.5_ (approximately 0.55).

Table 3 presents crude and adjusted HRs per IQR increase of NO_2_ (for the Montreal sub-cohort), O_3_, and PM_2.5_ levels at the birth residential postal code. Crude HRs for exposure at the birth residential postal code HRs were positive for NO_2_ (1.024, 95% CI: (1.015–1.034)) and PM_2.5_ (HR per IQR increase 1.056, 95% CI: (1.055–1.058)). Controlling for the ordinal number of exacerbation, year of birth, sex, as well as indices of social and material deprivation in a model stratified for age groups removed the association with NO_2_ (HR per IQR increase: 1.001, 95% CI: (0.995–1.005)) whereas only a slight reduction of the point estimates was observed for PM_2.5_ (HR per IQR increase 1.051, 95% CI: (1.049–1.053)). The HRs for O_3_ all presented no association with exacerbations. No significant effect modification was found with deprivation or sex.

In time dependent models, associations between asthma exacerbation and NO_2_ (Montreal sub-cohort), O_3_, and PM_2.5_ levels were respectively 1.024 (1.015–1.034), 0.996 (0.984–1.009) and 1.056 (1.055–1.058) per increase in IQR. Controlling for the year of birth, sex as well as social and material deprivation in gap time models stratified for age groups decreased the associations with time dependent air pollution exposures for each air pollutant (Table 4). Whereas this induced a marked decrease for NO_2_ (HR per IQR 1.190 95% CI 1.152–1.226 to 1.100 95% CI 1.063–1.135), the reduction was of a lesser magnitude for PM_2.5_ and O_3_. Controlling for the ordinal number of the exacerbation did not induce important changes in any of the HRs or their confidence intervals. As mentioned in the Methods, throughout the study, we used 2001–2006 PM_2.5_ levels as PM_2.5_ exposure, so the time dependent exposure only varies for subjects that moved during follow-up Furthermore, a significant effect modification was found between NO_2_ exposure and sex. The risk of exacerbation associated with NO_2_ seems to increase for girls (HR of interaction term per IQR 1.057 95% CI 1.005–1.112).

Associations with exposure at the birth residential postal code adjusted indirectly for SHS (for the Montreal sub-cohort) resulted in an increase in the variability of each point estimate. However, the indirect adjustment for SHS suggests that SHS does not bias the estimates from the main analyses as the HRs indirectly adjusted for SHS were very similar to unadjusted HRs (Table S1). Furthermore, the exclusion of regions of the province where the use of health services may be under-reported (respectively, for analyses with PM_2.5_ and O_3_, an exclusion of 8% and 6% of the children) provided similar results to the ones presented in Table 3 and Table 4 for the exposure at birth and time dependent exposures (Table S2). HRs per IQR increase for analyses restricted to non-movers (Table S3), decreased for O_3_ and PM_2.5_ but increased for NO_2_. However, since the population was reduced, a widening of the confidence interval was noted.

## 4. Discussion

We followed asthmatic children in Quebec over a period of fifteen years and found positive and significant associations between severe asthma exacerbation in children, and time dependent PM_2.5_, NO_2_ and O_3_ levels. Even though associations were noted with long-term time dependent NO_2_ and O_3_ levels, no association was found with exposure to these pollutants at the birth residential postal code. PM_2.5_ was the only air pollutant assessed for which exposure at the birth was linked to an increase risk of exacerbations during follow-up. Since each residential postal code was assigned the 2002–2006 average PM_2.5_ levels throughout the follow-up, the exposure only varied for children that moved outside of a 10 km × 10 km PM_2.5_ grid cell during their follow-up. Thus, it is particularly hard to disentangle the effect of long-term time dependent PM_2.5_ exposure and exposure at birth. These findings suggest, within the limitations of this study, that exposure at birth to NO_2_ and O_3_, at the level encountered in this study, are not linked to asthma exacerbations. Thus these findings do not support, for the two aforementioned pollutants, that in utero exposure would hinder children lung development in later life or that exposure in the first year of life would remodel immature airways [40,41]. However, they suggest that prolonged exposure to NO_2_, PM_2.5_ and O_3_ is mainly related to exacerbation of asthma.

To our knowledge, only one study assessed the association between long-term exposure to O_3_ and PM_2.5_ and asthma exacerbation in children already diagnosed with asthma [24]. This study was conducted on asthmatic children, aged between 0 and 17 years old, living in California. Odds ratios (ORs) linking follow-up specific air pollution levels and asthma hospitalization or emergency room visits in the previous year were 1.35 (95% CI 0.85–2.14) per 10 ppb increase of O_3_ and 1.09 (95% CI 0.47–2.50) per 10 µg/m^3^ of PM_2.5_. While reported associations with PM_2.5_ are similar to the ones presented in our study, the association with O_3_ is higher. This could possibly be explained by the large uncertainty around the point estimates or the much higher O_3_ levels encountered in the California study.

A few studies also assessed the effect of long-term exposure to air pollutants on asthma events in children, regardless of their asthmatic status. A cohort study conducted in the Netherlands [42] reported odds ratios with wheeze for two years old children, of 1.16 (95% CI 0.98–1.36) per 10.6 µg/m^3^ of NO_2_ and 1.20 (95% CI 0.99–1.46) per 3.3 µg/m^3^ of PM_2.5_. In Germany, Morgenstern et al. 2007 reported similar odds ratios with wheeze for the same age-group (OR for NO_2_: 1.09, 95% CI 0.90–1.31 per 10.6 µg/m^3^; OR for PM_2.5_: 1.10, 95% CI 0.96–1.25, per 1.04 µg/m^3^) [43]. A cross-sectional study [44] reported associations of 2.94 (95% CI 0.85–10.18 per 17.6 µg/m^3^ of NO_2_). Whereas associations with PM_2.5_ are in the same range as the ones reported here, those with NO_2_ are considerably higher. The disparities with our results could be due to the fact that we restricted our analyses to children already diagnosed with asthma and that we considered exacerbations as asthma related hospital admissions or emergency department visits, which are more severe compared to milder events such as wheezing. This difference could also be explained by other factors such as ways that the exposure was estimated.

Our study has numerous strengths. First, since asthmatic children were retrieved from a cohort that nearly covers the entire population of the province of Quebec, it virtually eliminates the likelihood of a selection bias. The resident of the province of Québec have access to a universal and free healthcare. Second, the large population and the length of the follow-up provide important statistical power to detect small effects, which is what was expected. Third, the NO_2_ and O_3_ models had a sufficiently small resolution to enable the assessment of the geographical dispersion of air pollutants at a relatively small scale in urban regions. Finally, we were able to consider the residential history of the children and thus assess the temporal variation of their exposures.

Still, there are several limitations to the present study. First, the definition of asthma exacerbation varies in the scientific literature (hospital admissions, use of asthma related medication, self-reported symptoms like wheeze, etc.) [45]. The fact that we used a definition based on health care service use (hospital admissions and emergency department visits) instead of intake of asthma medication could lead us to identify predominantly more severe cases of exacerbations. Furthermore, information on health care service use is not a perfect proxy of the exacerbation of asthma since several factors, such as accessibility to health care infrastructure, asthma management and socio-demographic characteristics can also modulate health care service use [46,47,48]. However associations between exposure to traffic and asthma exacerbations have been reported to be of a similar magnitude when exacerbations were defined by health care service use and self-report symptoms [24,49]. Furthermore, crude and adjusted time dependent exposure models for social and material deprivation provided similar results.

Second, asthma exacerbations are hard to delimitate through time in medico-administrative databases [50]. In order to prevent counting the same exacerbation more than once, a 30-day buffer period was used after each event. During this period, participants were considered not at risk of having an asthma exacerbation. Thirty days is a long period that leads to a conservative estimate of the number of recurring events. However, previous analyses (data not shown) showed that numbers of asthmatic children who had an exacerbation using a 30 days buffer or a 14 days buffer period are very similar (respectively, 21.6% and 22.8%).

A third limitation is that individual exposure was assessed using models instead of being measured throughout the follow up. Therefore the quality of associations is dependent on the quality of the exposure models. Furthermore, we had to assign individual exposures according to the pollutant level at the centroid of the postal code that encompassed each child residence. Since postal codes have a much smaller area in urban regions than rural regions, higher degree of imprecision could be associated with the exposure of children living in rural areas than those living in urban centers. This could lead to measurement errors in pollution exposure especially for pollutants that have a high spatial variability such as NO_2_. However, NO_2_ exposure was assessed for a densely populated area, i.e., for Montreal, where postal codes are small geographic units with an average area of 10,038 m^2^. A second source of imprecision in our exposure estimates resides in the resolution of each model. While exposure was estimated with imprecisions, we expect the measurement errors to be predominantly of Berkson type (i.e., the error is independent from the observed variable), which would induce little to no bias in point estimates [51]. Of the three pollutants, only the NO_2_ and O_3_ models have the small scale resolution needed to assess the urban variability of exposure. Although a PM_2.5_ model with a finer resolution would have been preferable, the local spatial variability associated with this air pollutant is much smaller than with NO_2_ levels. Moreover, throughout the follow-up (from 1996 to 2011) we assigned a 2001–2006 average concentration at each postal code. Therefore we could not take into account the temporal variation in PM_2.5_ levels. However, for the North American east coast, only a small decrease in trends of satellite derived time dependent PM_2.5_ levels was observed for the 1998−2012 period [27]. We also are likely to have overestimated yearly exposure to O_3_ by using summer levels. Lastly, the exposure assessment does not take into account of activity patterns or indoor infiltration of pollutants, which could lead to measurement errors. Furthermore, in the summer time, the use of air conditioning could also influence exposure of subjects [52]. Since the prevalence of air conditioning is lower in more deprived neighbourhoods this could induce an information bias. Nonetheless by controlling for material and social deprivation we reduce the likelihood of said bias even though residual confounding is still possible since we used an ecological variable that does not necessarily reflect perfectly individual deprivation status.

A fourth limitation is that residential exposure was assessed according to self-reported addresses with a mandatory update every four years. This self-report association could induce measurement error in air pollution exposure for people that moved during follow-up. Yet, analyses on non-movers presented similar association than those of the overall population.

Finally, as in most cohort studies based on administrative health databases, we lacked information on several risk factors at the individual level (e.g., socioeconomic status and smoking). To mitigate this deficiency we performed a sensitivity analysis with Montreal children in order to estimate the potential bias associated with missing individual information for exposure to secondhand smoke. The results suggest that the point estimates are not strongly biased by secondhand smoke. Other important risk factors linked to asthma exacerbation in asthmatic children, such as family history of asthma, could not be controlled in our analyses.

## 5. Conclusions

Our results showed consistent associations between time dependent exposure to PM_2.5_, O_3_ or NO_2_ and asthma exacerbation in asthmatic children. However, our results with O_3_ and NO_2_ do not support the hypothesis that residential exposure to air pollution at birth is associated with risk of increased asthma exacerbation.

## Figures and Tables

**Table 1:** Descriptive statistics of asthmatic study participants, for the Quebec cohort and the Montreal subgroup, 1996–2011.

Characteristics	Quebec	Montreal
Number of subjects	162,752	39,065
Male (%)	51.2	52.7
Number of participants with ≥1 exacerbation	35,229	10,529
Number of exacerbations	77,450	25,798
Pampalon material deprivation index (%)		
1 (Least deprived)	19.06	18.87
2	19.95	22.32
3	19.75	18.40
4	20.06	18.66
5 (Most Deprived)	21.17	21.76
Pampalon social deprivation index (%)		
1 (Least deprived)	18.81	17.89
2	19.71	14.48
3	19.59	16.59
4	20.67	25.93
5 (Most Deprived)	21.22	25.10
Follow-up (person years)	1,020,280	255,070
Number of person-year per age-group (%)		
<1	12.03	14.71
1–5	52.70	55.30
6–13	35.27	29.99

**Table 2:** Distributions of estimated annual average concentrations of NO_2_, PM_2.5_ and O_3_ at both the time dependent and the birth residential postal code.

Exposure	Pollutants	Minimum	25%	50%	75%	Maximum	Interquartile Range	Mean
**Birth**	**NO_2_** ^a^	6.49	12.93	15.12	17.88	31.15	4.95	15.51
	**O_3_** ^b^	18.55	28.03	29.88	31.88	38.92	3.85	29.78
	**PM_2.5_** ^c^	4.42	12.59	13.85	14.4	14.85	1.82	13.11
**Time dependent**	**NO_2_** ^a^	6.08	12.42	14.68	17.42	31.15	4.99	15.04
	**O_3_** ^b^	16.19	29.2	30.8	32.42	38.92	3.23	30.57
	**PM_2.5_** ^c^	4.42	12.59	13.85	14.4	14.85	1.82	13.11

^a^ Restricted to the Montreal sub-cohort and for the years 1996–2006 (in ppb); ^b^ for the years 1999 to 2010 (in ppb); ^c^ for the years 1996 to 2011 (in µg/m^3^).

**Table 3:** Associations between asthma exacerbation and an increase in interquartile range of air pollutant levels at the birth residential postal code, from gap time models ^a^.

Pollutants	Sample Size	Interquartile Range	Hazard Ratios (95% CI)		
			Crude	Model 1 ^b^	Model 2 ^c^
**NO_2_** ^a^	39,110	4.95 ppb	1.024 (1.015–1.034)	0.995 (0.985–1.005)	1.001 (0.995–1.005)
**O_3_^d^**	108,107	3.85 ppb	0.996 (0.984–1.009)	0.992 (0.981–1.001)	0.995 (0.984–1.040)
**PM_2.5_^e^**	153,007	1.82 µg/m^3^	1.056 (1.055–1.058)	1.057 (1.055–1.058)	1.051 (1.049–1.053)

^a^ Restricted to the Montreal sub-cohort, for the years 1996 to 2006; ^b^ associations stratified by age-group and adjusted for year of birth, sex and indices of social and material deprivation; ^c^ associations stratified by age-group and adjusted for year of birth, sex, ordinal number of exacerbation and indices of social and material deprivation; ^d^ for the years 1999 to 2010; ^e^ for the years 1996 to 2011.

**Table 4:** Associations between asthma exacerbation and an increase in time dependent air pollutants levels at the residence, from Cox gap time models ^a^.

Pollutants	Sample Size	Interquartile Range	Hazard Ratios (95% CI)		
			Crude	Model 1 ^b^	Model 2 ^c^
**NO_2_** ^a^	39,110	4.99 ppb	1.190 (1.152–1.226)	1.100 (1.063–1.135)	1.095 (1.058–1.131)
**O_3_^d^**	108,107	3.23 ppb	1.069 (1.059–1.080)	1.043 (1.025–1.061)	1.052 (1.037–1.066)
**PM_2.5_^e^**	153,007	1.82 µg/m^3^	1.029 (1.022–1.035)	1.026 (1.019–1.032)	1.025 (1.017–1.031)

^a^ Restricted to the Montreal sub-cohort, for the years 1996 to 2006; ^b^ associations stratified by age-group and adjusted for year of birth, sex and indices of social and material deprivation; ^c^ associations stratified by age-group and adjusted for year of birth, sex, ordinal number of exacerbation and indices of social and material deprivation; ^d^ for the years 1999 to 2010; ^e^ for the years 1996 to 2011.

## Supplementary Materials

The following are available online at www.mdpi.com/1660-4601/13/8/771/s1. Table S1. Indirect adjustment for second hand smoke for associations between asthma exacerbation and an interquartile range increase in air pollutant levels at the birth address, form gap time models; Table S2. Associations between asthma exacerbation and an interquartile range increase in air pollutants levels at the home address form gap time models excluding some health regions of Quebec; Table S3. Associations between asthma exacerbation and an interquartile range increase in air pollutant levels at the birth address in non-movers, form gap time models.
