# Supplementary material for: Severe and Moderate Asthma Exacerbations in Asthmatic Children and Exposure to Ambient Air Pollutants
Source: Int J Environ Res Public Health. 2016 Aug 1;13(8):771. doi: 10.3390/ijerph13080771 (PMC4997457; doi:10.3390/ijerph13080771)
Supplement: Supplementary file 1 [file ijerph-13-00771-s001.pdf]

Supplementary Materials: Severe and Moderate Asthma Exacerbations in Asthmatic Children and Exposure to Ambient Air Pollutants

Louis-Francois Tétreault, Marieve Doucet, Philippe Gamache, Michel Fournier, Allan Brand, Tom Kosatsky and Audrey Smargiassi

Table S1. Indirect adjustment for second hand smoke for associations between asthma exacerbation and an interquartile range increase in air pollutant levels at the birth address, form gap time models [a,b].

Pollutants	Sample Size	Interquartile Range	Hazard Ratios (95% CI)	
			Montreal Sub-Cohort	Indirect Adjustment for Secondhand Smoke
NO_2 [c]	39,110	4.95 pp	1.001 (0.995–1.005)	1.000 (0.994–1.006)
O_3 [d]	25,054	3.85 pp	0.980 (0.935–1.026)	0.980 (0.934–1.028)
$PM_{2.5}$ [c]	39,110	1.82 μg/m³	1.019 (0.998–1.040)	1.017 (0.993–1.041)

[a] Restricted to the Montreal sub-cohort; [b] associations stratified by age-group and adjusted for year of birth, sex, ordinal number of exacerbation and indices of social and material deprivation; [c] for the years 1996 to 2006 [d] for the years 1999 to 2006.

Table S2. Associations between asthma exacerbation and an interquartile range increase in air pollutants levels at the home address form gap time models excluding some health regions of Quebec [a,b].

Pollutants	Sample Size	Exposure at Birth		Follow-Up Specific Exposure	
		Interquartile Range	Hazard Ratios (95% CI)	Interquartile Range	Hazard Ratios (95% CI)
O_3 [c]	101,760	3.85 ppb	0.999 (0.976–1.023)	3.23 ppb	1.055 (1.043–1.072)
$PM_{2.5}$ [d]	140,766	1.82 μg/m³	1.021 (1.012–1.027)	1.82 μg/m³	1.026 (1.017–1.031)

[a] Associations stratified by age-group and adjusted for year of birth, sex, ordinal number of exacerbation and indices of social and material deprivation; [b] the province of Quebec without the following social and healthcare regions: *North of Quebec, the North Shore, the Gaspésie and the Magdalene;* [c] for the years 1999 to 2010; [d] for the years 1996 to 2011.

Table S3. Associations between asthma exacerbation and an interquartile range increase in air pollutant levels at the birth address in non-movers, form gap time models [a].

Pollutants	Sample Size	Interquartile Range	Hazard Ratios (95% CI)
NO_2 [b]	4238	4.99 ppb	1.030 (0.971–1.092)
O_3 [c]	12,466	3.23 ppb	0.973 (0.929–1.019)
$PM_{2.5}$ [d]	18,304	1.82 μg/m³	1.026 (1.012–1.039)

[a] Associations stratified by age-group and adjusted for year of birth, sex, ordinal number of exacerbation and indices of social and material deprivation; [b] restricted to the Montreal sub-cohort, for the years 1996 to 2006 [c] for the years 1999 to 2010 [d] for the years 1996 to 2011.

© 2016 by the authors; licensee MDPI, Basel, Switzerland. This article is an open access article distributed under the terms and conditions of the Creative Commons by Attribution (CC-BY) license (http://creativecommons.org/licenses/by/4.0/).
